# A tetra­nuclear cobalt(III) cluster with 2-(hydroxy­meth­yl)pyridine ligands

**DOI:** 10.1107/S1600536810015369

**Published:** 2010-04-30

**Authors:** Fang-Ming Wang, Chang-Sheng Lu, Yi-Zhi Li, Qing-Jin Meng

**Affiliations:** a State Key Laboratory of Coordination Chemistry, Nanjing National Laboratory of Microstructures, School of Chemistry and Chemical Engineering, Nanjing University, Nanjing 210023, People’s Republic of China

## Abstract

In the title compound, tetra­kis[μ_3_-(2-pyrid­yl)methano­lato]tetra­kis[bromido(methanol)cobalt(III)] tetra­bromide 2-(hydroxy­meth­yl)pyridine tetra­solvate dihydrate,  [Co_4_Br_4_(C_6_H_6_NO)_4_(CH_3_OH)_4_]Br_4_·4C_6_H_7_NO_4_·2H_2_O, the cation comprises a [Co_4_O_4_] cubane-type core (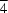 symmetry). The four Co^III^ ions and bridging O atoms from four (2-pyrid­yl)methano­late anions are located at alternating vertices of the cube, with bromide ions and methanol ligands on the exterior of the core, completing a distorted octa­hedral geometry. The structure is stablized by inter­molecular O—H⋯Br and O—H⋯O inter­actions.

## Related literature

For related structures and magnetic properties, see: Tong *et al.* (2002 ▶); Yang *et al.* (2002 ▶); Zhao *et al.* (2004 ▶). 
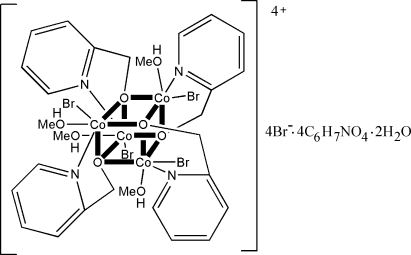


## Experimental

### 

#### Crystal data

[Co_4_Br_4_(C_6_H_6_NO)_4_(CH_4_O)_4_]Br_4_·4C_6_H_7_NO_4_·2H_2_O
*M* *_r_* = 1908.10
Tetragonal, 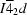

*a* = 16.5302 (6) Å
*c* = 29.875 (2) Å
*V* = 8163.3 (8) Å^3^
*Z* = 4
Mo *K*α radiation
μ = 4.77 mm^−1^
*T* = 291 K
0.30 × 0.24 × 0.22 mm

#### Data collection

Bruker SMART APEX CCD area-detector diffractometer
Absorption correction: multi-scan (*SADABS*; Bruker, 2000 ▶) *T*_min_ = 0.265, *T*_max_ = 0.350
21461 measured reflections
4018 independent reflections
3180 reflections with *I* > 2σ(*I*)
*R*_int_ = 0.056

#### Refinement

*R*[*F* ^2^ > 2σ(*F* ^2^)] = 0.052
*wR*(*F* ^2^) = 0.113
*S* = 0.99
4018 reflections
200 parameters
H-atom parameters constrained
Δρ_max_ = 0.75 e Å^−3^
Δρ_min_ = −0.56 e Å^−3^
Absolute structure: Flack (1983 ▶), 1822 Friedel pairs
Flack parameter: 0.025 (18)

### 

Data collection: *SMART* (Bruker, 2000 ▶); cell refinement: *SAINT* (Bruker, 2000 ▶); data reduction: *SAINT*; program(s) used to solve structure: *SHELXTL* (Sheldrick, 2008 ▶); program(s) used to refine structure: *SHELXTL*; molecular graphics: *SHELXTL*; software used to prepare material for publication: *SHELXTL*.

## Supplementary Material

Crystal structure: contains datablocks I, global. DOI: 10.1107/S1600536810015369/bx2274sup1.cif

Structure factors: contains datablocks I. DOI: 10.1107/S1600536810015369/bx2274Isup2.hkl

Additional supplementary materials:  crystallographic information; 3D view; checkCIF report

## Figures and Tables

**Table 1:** Hydrogen-bond geometry (Å, °)

*D*—H⋯*A*	*D*—H	H⋯*A*	*D*⋯*A*	*D*—H⋯*A*
O2—H2*B*⋯Br1^i^	0.97	2.54	3.217 (5)	127
O3—H3*A*⋯O3^ii^	0.96	2.31	3.030 (9)	132
O3—H3*A*⋯O4^iii^	0.96	2.57	3.370 (11)	141

Symmetry codes: (i) 
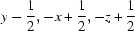
; (ii) 
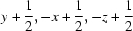
; (iii) 
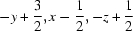
.

**Fig. 1.**
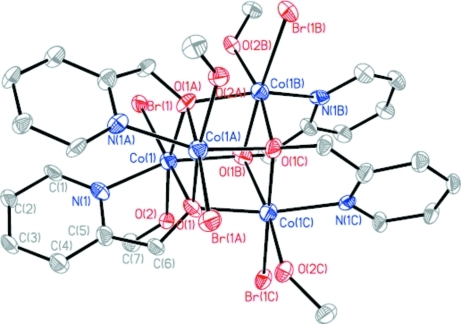
A view of the molecular structure of the cationic cubane core.

## supplementary crystallographic information

### Comment

There have characterized many polynuclear oxide-bridged metal complexes which having a cubane-type structural geometry, because of their relevance to multi-electron transfer centers in biological systems, and to their interesting magnetic and optical properties, as well as to their potential relevance to inorganic solids.

In this work, we synthesized a new tetranuclearCo(III) cluster [Co(hmp)(MeOH)Br]_4_Br_4_.4Hhmp.2H_2_O which comprise a cationic "cubane"-type core (1), where Hhmp is 2-(hydroxymethyl)pyridine. The molecular structure of the cationic cubane core of (1) is shown in Fig. 1. The four cobalt ions and bridging hydroxy group oxygen atoms from four (2-pyridyl)methanolate anions are located at alternating vertices of a cube, with (2-pyridyl)methanolate anion, bromine ion and methanol ligand on the exterior of the core. Furthermore, the three-dimensional supramolecularstructure is stabilized by intramolecular and intermolecular hydrogen bonds. The hydrogen-bonding distances are 3.217 (5) Å (O2–H2B···Br1), 3.030 (9) Å (O3–H3A···O3) and 3.370 (11)Å (O3–H3A···O4), Table 1.

### Experimental

Compound [Co(hmp)(MeOH)Br]_4_Br_4_.4Hhmp.2H_2_O was synthesized as the process shown in reference (Yang *et al.*, 2002). A mixture of CoBr_2_.6H_2_O (0.327 g, 1 mmol), Hhmp (0.109 g, 1 mmol), and NaOMe (0.054 g, 1 mmol) in 10 ml of MeOH was refluxed for 30 min. The resulting solution was filtered when it was still hot. Purple crystals suitable for X-ray analysis were obtained from the filtrate after several days.

### Refinement

All H atoms were fixed geometrically and were treated as riding on their parent C atoms, with C–H distances in the range of 0.93–0.96 Å, and with *U*_iso_(H) = 1.2*U*_eq_(parent atom), or *U*_iso_(H) = 1.5*U*_eq_(C_methyl_).

### Crystal data

**Table:**

[Co_4_Br_4_(C_6_H_6_NO)_4_(CH_4_O)_4_]Br_4_·4C_6_H_7_NO_4_·2H_2_O	*D*_x_ = 1.553 Mg m^−^^3^
*M**_r_* = 1908.10	Mo *K*α radiation, λ = 0.71073 Å
Tetragonal, *I*4_2_*d*	Cell parameters from 4154 reflections
Hall symbol: I -4 2bw	θ = 2.2–23.1°
*a* = 16.5302 (6) Å	µ = 4.77 mm^−^^1^
*c* = 29.875 (2) Å	*T* = 291 K
*V* = 8163.3 (8) Å^3^	Block, purple
*Z* = 4	0.30 × 0.24 × 0.22 mm
*F*(000) = 3760	

### Data collection

**Table:**

Bruker SMART APEX CCD area-detector diffractometer	4018 independent reflections
Radiation source: sealed tube	3180 reflections with *I* > 2σ(*I*)
graphite	*R*_int_ = 0.056
phi and ω scans	θ_max_ = 26.0°, θ_min_ = 2.2°
Absorption correction: multi-scan (*SADABS*; Bruker, 2000)	*h* = −15→20
*T*_min_ = 0.265, *T*_max_ = 0.350	*k* = −20→17
21461 measured reflections	*l* = −32→36

### Refinement

**Table:**

Refinement on *F*^2^	Secondary atom site location: difference Fourier map
Least-squares matrix: full	Hydrogen site location: inferred from neighbouring sites
*R*[*F*^2^ > 2σ(*F*^2^)] = 0.052	H-atom parameters constrained
*wR*(*F*^2^) = 0.113	*w* = 1/[σ^2^(*F*_o_^2^) + (0.0635*P*)^2^] where *P* = (*F*_o_^2^ + 2*F*_c_^2^)/3
*S* = 0.99	(Δ/σ)_max_ < 0.001
4018 reflections	Δρ_max_ = 0.75 e Å^−^^3^
200 parameters	Δρ_min_ = −0.56 e Å^−^^3^
0 restraints	Absolute structure: Flack (1983), 1822 Friedel pairs
Primary atom site location: structure-invariant direct methods	Flack parameter: 0.025 (18)

### Special details

**Table:**

Geometry. All esds (except the esd in the dihedral angle between two l.s. planes) are estimated using the full covariance matrix. The cell esds are taken into account individually in the estimation of esds in distances, angles and torsion angles; correlations between esds in cell parameters are only used when they are defined by crystal symmetry. An approximate (isotropic) treatment of cell esds is used for estimating esds involving l.s. planes.
Refinement. Refinement of *F*^2^ against ALL reflections. The weighted *R*-factor wR and goodness of fit *S* are based on *F*^2^, conventional *R*-factors *R* are based on *F*, with *F* set to zero for negative *F*^2^. The threshold expression of *F*^2^ > σ(*F*^2^) is used only for calculating *R*-factors(gt) etc. and is not relevant to the choice of reflections for refinement. *R*-factors based on *F*^2^ are statistically about twice as large as those based on *F*, and *R*- factors based on ALL data will be even larger.

### Fractional atomic coordinates and isotropic or equivalent isotropic displacement parameters (Å^2^)

**Table:**

	*x*	*y*	*z*	*U*_iso_*/*U*_eq_	Occ. (<1)
Br1	−0.05719 (4)	0.72378 (5)	0.20269 (3)	0.04360 (19)	
Br2	0.27436 (4)	0.77454 (4)	0.29686 (2)	0.04162 (18)	
C1	0.0376 (4)	0.6172 (5)	0.1104 (3)	0.0415 (17)	
H1	−0.0035	0.6554	0.1126	0.050*	
C2	0.0725 (5)	0.6005 (5)	0.0696 (2)	0.0423 (18)	
H2	0.0556	0.6285	0.0443	0.051*	
C3	0.1331 (5)	0.5422 (5)	0.0658 (2)	0.0417 (18)	
H3	0.1564	0.5311	0.0381	0.050*	
C4	0.1584 (5)	0.5003 (5)	0.1042 (3)	0.0453 (18)	
H4	0.1974	0.4600	0.1021	0.054*	
C5	0.1241 (5)	0.5198 (5)	0.1460 (2)	0.0422 (17)	
C6	0.1358 (5)	0.4721 (5)	0.1851 (2)	0.0385 (16)	
H6A	0.1356	0.4156	0.1763	0.046*	
H6B	0.1891	0.4840	0.1969	0.046*	
C7	0.1841 (5)	0.7172 (5)	0.1922 (3)	0.0460 (18)	
H7A	0.2247	0.6876	0.1760	0.069*	
H7B	0.2096	0.7567	0.2110	0.069*	
H7C	0.1488	0.7439	0.1714	0.069*	
C8	0.5885 (5)	−0.0934 (5)	0.1084 (2)	0.0432 (18)	
H8	0.5728	−0.1472	0.1111	0.052*	
C9	0.6092 (5)	−0.0630 (5)	0.0676 (3)	0.0415 (17)	
H9	0.6083	−0.0967	0.0426	0.050*	
C10	0.6315 (5)	0.0173 (5)	0.0626 (3)	0.0468 (19)	
H10	0.6441	0.0378	0.0345	0.056*	
C11	0.6350 (4)	0.0673 (5)	0.1005 (3)	0.0454 (19)	
H11	0.6512	0.1210	0.0978	0.055*	
C12	0.6141 (5)	0.0361 (5)	0.1423 (3)	0.0446 (18)	
C13	0.6198 (5)	0.0845 (5)	0.1836 (3)	0.0458 (19)	
H13A	0.6736	0.0803	0.1964	0.055*	
H13B	0.6085	0.1409	0.1774	0.055*	
Co1	0.02308 (6)	0.59361 (6)	0.21302 (3)	0.0396 (2)	
N1	0.0641 (4)	0.5764 (3)	0.1481 (2)	0.0367 (14)	
N2	0.5908 (4)	−0.0430 (4)	0.1466 (2)	0.0407 (14)	
O1	0.0801 (3)	0.4821 (3)	0.21907 (16)	0.0415 (11)	
O2	0.1369 (3)	0.6610 (3)	0.21993 (16)	0.0384 (11)	
H2B	0.1741	0.6162	0.2238	0.046*	
O3	0.5652 (3)	0.0547 (3)	0.21175 (16)	0.0445 (12)	
H3A	0.5927	0.0323	0.2372	0.053*	
O4	0.5585 (6)	0.7840 (6)	0.2123 (3)	0.044 (2)	0.50
H4A	0.5964	0.7521	0.2297	0.053*	0.50
H4B	0.5115	0.7846	0.2318	0.053*	0.50

### Atomic displacement parameters (Å^2^)

**Table:**

	*U*^11^	*U*^22^	*U*^33^	*U*^12^	*U*^13^	*U*^23^
Br1	0.0435 (4)	0.0479 (4)	0.0394 (4)	0.0080 (3)	0.0066 (3)	0.0131 (3)
Br2	0.0410 (4)	0.0412 (4)	0.0427 (4)	−0.0152 (3)	0.0104 (3)	0.0128 (3)
C1	0.037 (4)	0.046 (4)	0.041 (4)	0.014 (3)	0.018 (3)	0.001 (3)
C2	0.042 (4)	0.050 (4)	0.035 (4)	0.008 (3)	0.010 (3)	0.014 (3)
C3	0.047 (4)	0.042 (4)	0.035 (4)	0.011 (3)	0.020 (3)	0.002 (3)
C4	0.049 (4)	0.043 (4)	0.044 (4)	0.009 (3)	0.012 (4)	0.006 (3)
C5	0.049 (4)	0.041 (4)	0.036 (4)	0.004 (3)	0.008 (3)	0.010 (3)
C6	0.037 (4)	0.041 (4)	0.037 (4)	0.012 (3)	0.013 (3)	0.010 (3)
C7	0.048 (4)	0.039 (4)	0.051 (5)	−0.003 (3)	0.007 (3)	0.010 (4)
C8	0.046 (4)	0.044 (4)	0.040 (4)	0.023 (3)	0.010 (3)	0.015 (3)
C9	0.042 (4)	0.045 (4)	0.038 (4)	0.011 (3)	0.006 (3)	0.018 (3)
C10	0.051 (5)	0.054 (5)	0.035 (4)	−0.009 (4)	−0.011 (3)	0.019 (4)
C11	0.038 (4)	0.049 (5)	0.050 (5)	0.014 (3)	0.013 (3)	0.008 (4)
C12	0.044 (4)	0.044 (4)	0.046 (4)	−0.020 (3)	0.008 (3)	−0.006 (3)
C13	0.054 (5)	0.031 (4)	0.052 (5)	0.007 (3)	0.005 (4)	−0.008 (3)
Co1	0.0400 (5)	0.0405 (5)	0.0384 (6)	0.0002 (4)	0.0028 (4)	0.0029 (4)
N1	0.037 (3)	0.029 (3)	0.044 (3)	−0.009 (2)	0.013 (3)	0.002 (2)
N2	0.040 (3)	0.038 (3)	0.044 (4)	−0.001 (3)	0.012 (3)	0.011 (3)
O1	0.041 (3)	0.042 (3)	0.041 (3)	0.004 (2)	0.018 (2)	0.005 (2)
O2	0.036 (3)	0.044 (3)	0.035 (3)	0.0024 (19)	0.000 (2)	0.010 (2)
O3	0.052 (3)	0.052 (3)	0.029 (3)	−0.012 (2)	−0.014 (2)	−0.008 (2)
O4	0.050 (6)	0.040 (5)	0.041 (6)	−0.001 (5)	0.009 (5)	0.008 (5)

### Geometric parameters (Å, °)

**Table:**

Br1—Co1	2.5467 (12)	C9—C10	1.386 (11)
C1—C2	1.376 (10)	C9—H9	0.9300
C1—N1	1.384 (10)	C10—C11	1.403 (12)
C1—H1	0.9300	C10—H10	0.9300
C2—C3	1.394 (11)	C11—C12	1.394 (11)
C2—H2	0.9300	C11—H11	0.9300
C3—C4	1.405 (11)	C12—N2	1.370 (9)
C3—H3	0.9300	C12—C13	1.474 (11)
C4—C5	1.407 (10)	C13—O3	1.328 (10)
C4—H4	0.9300	C13—H13A	0.9700
C5—N1	1.366 (10)	C13—H13B	0.9700
C5—C6	1.422 (10)	Co1—O1^i^	2.043 (5)
C6—O1	1.381 (8)	Co1—N1	2.073 (6)
C6—H6A	0.9700	Co1—O1	2.079 (5)
C6—H6B	0.9700	Co1—O1^ii^	2.123 (5)
C7—O2	1.469 (9)	Co1—O2	2.196 (5)
C7—H7A	0.9600	O1—Co1^iii^	2.043 (5)
C7—H7B	0.9600	O1—Co1^ii^	2.123 (5)
C7—H7C	0.9600	O2—H2B	0.9700
C8—C9	1.362 (10)	O3—H3A	0.9601
C8—N2	1.415 (10)	O4—H4A	0.9700
C8—H8	0.9300	O4—H4B	0.9700
			
C2—C1—N1	119.4 (6)	N2—C12—C11	120.5 (7)
C2—C1—H1	120.3	N2—C12—C13	117.2 (7)
N1—C1—H1	120.3	C11—C12—C13	122.3 (7)
C1—C2—C3	120.8 (7)	O3—C13—C12	106.6 (7)
C1—C2—H2	119.6	O3—C13—H13A	110.4
C3—C2—H2	119.6	C12—C13—H13A	110.4
C2—C3—C4	119.2 (6)	O3—C13—H13B	110.4
C2—C3—H3	120.4	C12—C13—H13B	110.4
C4—C3—H3	120.4	H13A—C13—H13B	108.6
C3—C4—C5	119.5 (7)	O1^i^—Co1—N1	158.0 (2)
C3—C4—H4	120.3	O1^i^—Co1—O1	80.55 (19)
C5—C4—H4	120.3	N1—Co1—O1	79.1 (2)
N1—C5—C4	119.4 (7)	O1^i^—Co1—O1^ii^	79.5 (2)
N1—C5—C6	116.1 (6)	N1—Co1—O1^ii^	105.2 (2)
C4—C5—C6	123.1 (7)	O1—Co1—O1^ii^	80.5 (2)
O1—C6—C5	116.6 (6)	O1^i^—Co1—O2	89.86 (19)
O1—C6—H6A	108.1	N1—Co1—O2	82.9 (2)
C5—C6—H6A	108.1	O1—Co1—O2	93.03 (19)
O1—C6—H6B	108.1	O1^ii^—Co1—O2	168.29 (18)
C5—C6—H6B	108.1	O1^i^—Co1—Br1	101.00 (14)
H6A—C6—H6B	107.3	N1—Co1—Br1	99.94 (17)
O2—C7—H7A	109.5	O1—Co1—Br1	175.05 (15)
O2—C7—H7B	109.5	O1^ii^—Co1—Br1	95.13 (14)
H7A—C7—H7B	109.5	O2—Co1—Br1	91.68 (13)
O2—C7—H7C	109.5	C5—N1—C1	121.6 (6)
H7A—C7—H7C	109.5	C5—N1—Co1	112.1 (5)
H7B—C7—H7C	109.5	C1—N1—Co1	126.3 (5)
C9—C8—N2	119.9 (8)	C12—N2—C8	119.6 (6)
C9—C8—H8	120.0	C6—O1—Co1^iii^	130.8 (4)
N2—C8—H8	120.0	C6—O1—Co1	110.1 (4)
C8—C9—C10	121.0 (8)	Co1^iii^—O1—Co1	99.9 (2)
C8—C9—H9	119.5	C6—O1—Co1^ii^	113.7 (5)
C10—C9—H9	119.5	Co1^iii^—O1—Co1^ii^	98.49 (19)
C9—C10—C11	119.3 (7)	Co1—O1—Co1^ii^	98.7 (2)
C9—C10—H10	120.3	C7—O2—Co1	136.3 (4)
C11—C10—H10	120.3	C7—O2—H2B	102.3
C12—C11—C10	119.6 (8)	Co1—O2—H2B	99.6
C12—C11—H11	120.2	C13—O3—H3A	108.8
C10—C11—H11	120.2	H4A—O4—H4B	101.6
			
N1—C1—C2—C3	1.3 (12)	O2—Co1—N1—C1	−102.6 (6)
C1—C2—C3—C4	−0.1 (12)	Br1—Co1—N1—C1	−12.1 (6)
C2—C3—C4—C5	−2.0 (12)	C11—C12—N2—C8	0.3 (11)
C3—C4—C5—N1	3.0 (12)	C13—C12—N2—C8	−176.7 (7)
C3—C4—C5—C6	169.2 (8)	C9—C8—N2—C12	−0.1 (11)
N1—C5—C6—O1	6.7 (11)	C5—C6—O1—Co1^iii^	−146.0 (6)
C4—C5—C6—O1	−159.9 (7)	C5—C6—O1—Co1	−22.0 (8)
N2—C8—C9—C10	−1.0 (11)	C5—C6—O1—Co1^ii^	87.7 (7)
C8—C9—C10—C11	1.8 (12)	O1^i^—Co1—O1—C6	−150.0 (5)
C9—C10—C11—C12	−1.5 (11)	N1—Co1—O1—C6	21.6 (5)
C10—C11—C12—N2	0.5 (12)	O1^ii^—Co1—O1—C6	129.2 (4)
C10—C11—C12—C13	177.4 (7)	O2—Co1—O1—C6	−60.7 (5)
N2—C12—C13—O3	−30.4 (10)	Br1—Co1—O1—C6	101.3 (16)
C11—C12—C13—O3	152.6 (7)	O1^i^—Co1—O1—Co1^iii^	−9.59 (19)
C4—C5—N1—C1	−1.9 (10)	N1—Co1—O1—Co1^iii^	162.0 (3)
C6—C5—N1—C1	−169.1 (7)	O1^ii^—Co1—O1—Co1^iii^	−90.4 (2)
C4—C5—N1—Co1	179.5 (6)	O2—Co1—O1—Co1^iii^	79.8 (2)
C6—C5—N1—Co1	12.4 (8)	Br1—Co1—O1—Co1^iii^	−118.2 (15)
C2—C1—N1—C5	−0.2 (11)	O1^i^—Co1—O1—Co1^ii^	90.7 (2)
C2—C1—N1—Co1	178.1 (6)	N1—Co1—O1—Co1^ii^	−97.7 (2)
O1^i^—Co1—N1—C5	4.2 (9)	O1^ii^—Co1—O1—Co1^ii^	9.9 (3)
O1—Co1—N1—C5	−18.6 (5)	O2—Co1—O1—Co1^ii^	−179.97 (19)
O1^ii^—Co1—N1—C5	−95.5 (5)	Br1—Co1—O1—Co1^ii^	−18.0 (17)
O2—Co1—N1—C5	75.9 (5)	O1^i^—Co1—O2—C7	−160.9 (6)
Br1—Co1—N1—C5	166.4 (5)	N1—Co1—O2—C7	39.9 (6)
O1^i^—Co1—N1—C1	−174.3 (6)	O1—Co1—O2—C7	118.6 (6)
O1—Co1—N1—C1	163.0 (6)	O1^ii^—Co1—O2—C7	174.5 (9)
O1^ii^—Co1—N1—C1	86.1 (6)	Br1—Co1—O2—C7	−59.9 (6)

Symmetry codes: (i) −*y*+1/2, *x*+1/2, −*z*+1/2; (ii) −*x*, −*y*+1, *z*; (iii) *y*−1/2, −*x*+1/2, −*z*+1/2.

### Hydrogen-bond geometry (Å, °)

**Table:**

*D*—H···*A*	*D*—H	H···*A*	*D*···*A*	*D*—H···*A*
O2—H2B···Br1^iii^	0.97	2.54	3.217 (5)	127
O3—H3A···O3^iv^	0.96	2.31	3.030 (9)	132
O3—H3A···O4^v^	0.96	2.57	3.370 (11)	141

Symmetry codes: (iii) *y*−1/2, −*x*+1/2, −*z*+1/2; (iv) *y*+1/2, −*x*+1/2, −*z*+1/2; (v) −*y*+3/2, *x*−1/2, −*z*+1/2.
